# Supplementary material for: Identification of photoperiod-induced specific miRNAs in the adrenal glands of Sunite sheep (Ovis aries)
Source: Front Vet Sci. 2022 Jul 22;9:888207. doi: 10.3389/fvets.2022.888207 (PMC9354845; doi:10.3389/fvets.2022.888207)
Supplement: Supplementary file 1 [file Data_Sheet_1.ZIP › Supplementary file/miRDeep2 file.zip!/miRDeep2 file/Know_Structure/LP42A3/oar-mir-154a.pdf]

miRBase precursor : oar-mir-154a
Total read count : 1300
oar-miR-154a-5p read count : 13
oar-miR-154a-3p read count : 1283
remaining reads : 4

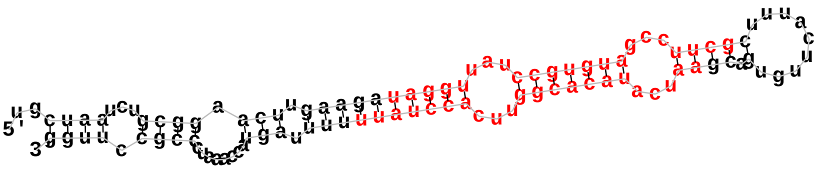

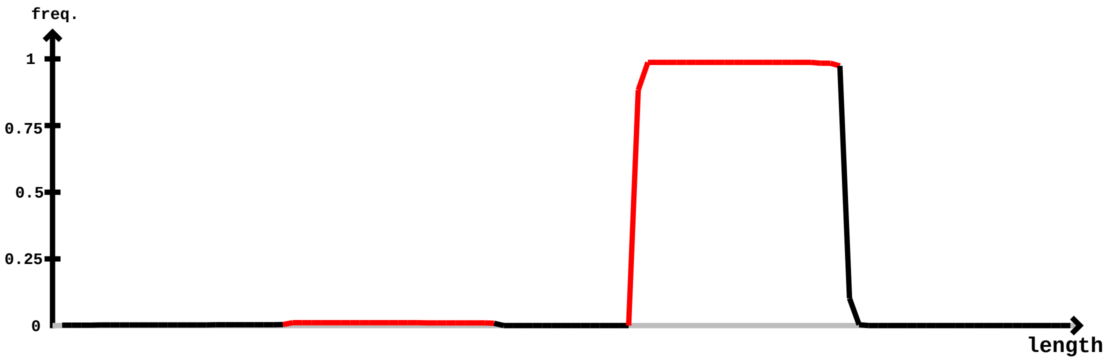

oar-miR-154a-5p

oar-miR-154a-3p

```
5'- ugcuaaucugcggaacuugaagauagguuauccguguagccuucgcuuuacuugugacgaaucauacacgguucaccuauuuuuuaguaccaaaucccgccuugg   -3'    exp
    .((......))((.(((.((((((((((...(((((((...(((((.........).))))...)))))))...))))))))))).))).)).............. reads    mm    sample
    ugcuaaucugcggaacuugaaga..................................................................................   2       0     seq
    ...uaaucugcggaacuugaaga..................................................................................   1       0     seq
    ...............cuugaagauagguuauccgugu....................................................................   1       0     seq
    ......................auagguuauccguguagccuucg............................................................   1       0     seq
    .......................uagguuauccguguagccuuc.............................................................   2       0     seq
    .......................uagguuauccguguagccuucg............................................................   10      0     seq
    ...........................................................aaucauacacgguucaccu...........................   4       0     seq
    ...........................................................aaucauacacgguucaccuau.........................   13      0     seq
    ...........................................................aaucauacacgguucaccuauu........................   1105    0     seq
    ...........................................................aaucauacacgguucaccuauuu.......................   26      0     seq
    ............................................................aucauacacgguucaccuauu........................   28      0     seq
    ............................................................aucauacacgguucaccuauuu.......................   103     0     seq
    ............................................................aucauacacgguucaccuauuuu......................   4       0     seq
```
